# Effect of dentin pretreatment on the resulting abrasive dentin wear

**DOI:** 10.1186/s12903-021-01648-3

**Published:** 2021-06-09

**Authors:** Blend Hamza, Marina Kazimi, Philipp Körner, Thomas Attin, Florian Just Wegehaupt

**Affiliations:** Clinic of Orthodontics and Pediatric Dentistry, Center of Dental Medicine, University of Zurich, Plattenstrasse 11, 8032 Zurich, Switzerland

**Keywords:** Abrasive dentine wear, Pretreatment, Toothbrushing

## Abstract

**Background:**

This study aims to investigate the influence of different dentin pretreatment procedures on the resulting abrasive dentin wear.

**Methods:**

Two groups (A, B) of 60 dentin samples each were prepared. Group A was brushed with an abrasive slurry (RDA = 85) and group B with a different abrasive slurry (RDA = 174). Four subgroups in each group (n = 15) were created (A1–A4) and (B1–B4). The subgroups were pretreated as follows: A1 + B1 with 1200-grit grinding paper, A2 + B2 with 1200- and 2000-grit papers, A3 + B3 with 1200-, 2000-, and 4000-grit papers, A4 + B4 with 1200-, 2000-, 4000-grit papers and with 1000 brushing strokes with a slurry of Elmex toothpaste. All samples were brushed for 25 min at 120 strokes/min. Abrasive dentin wear was measured for each sample profilometrically and the subgroups were compared with each other within the same group. Repeated measures one-way ANOVA was conducted to compare the subgroups and pairwise contrasts were estimated for multiple testing according to Tukey (α = 0.05).

**Results:**

The resulting abrasive dentin wear in group A ranged between 15.3 ± 3.4 µm and 17.3 ± 5.5 µm and between 20.3 ± 6.8 µm and 22.5 ± 2.6 µm in group B. No statistically significant difference was noticed between any subgroups within the same group (*p *˃ 0.05).

**Conclusions:**

Different dentin pretreatment procedures do not affect the resulting abrasive dentin wear independent of the RDA value of the employed abrasive slurry.

## Background

Abrasives have always been an essential component in toothpastes. They enhance the ability of toothpastes to remove plaque and stains from teeth surfaces [1]. However, their presence has also been connected to some hazardous effect on enamel and dentin, the so-called “abrasive dentin—or enamel—wear” [2]. Many studies investigating abrasive dentin wear—and its potential involvement in non-carious cervical lesions—have been carried out [3–6].

Many abrasion studies have been conducted on bovine dentin samples. In order to create a standardized baseline situation, these samples usually undergo a certain pretreatment procedure where they are ground using different grinding papers. While dentin samples were ground at 1200 grit in some studies [7, 8], other studies used dentin samples ground at 4000 grit [6, 9]. Additionally to grounding dentin samples, some studies also treated dentin samples with different slurries as a pretreatment step [10–12]. This additional step pretreats the samples in a way resembling the situation in the oral cavity, where no ground dentin surfaces are found and teeth are daily brushed with a toothpaste, and thus might result in a more clinically relevant situation.

It is well known that the abrasive dentin wear is attributed to a multitude of factors, and thus any modification of one or more of these factors would have an effect on the resulting wear [13]. This study was therefore carried out to investigate the influence of different dentin pretreatment procedures on the resulting abrasive dentin wear employing two abrasive slurries with different RDA values. The findings might help understanding the discrepancy between different laboratories regarding measuring abrasivity and elaborate the necessity of complex pretreatment procedures of the dentin samples in abrasion studies. The null hypothesis of this study was that there is no difference in the resulting abrasive dentin wear if the dentin samples have been prepared with different pretreatment procedures.

## Methods

Thirty bovine permanent incisors were used in this study. The incisors were numbered and divided into two groups; group A consisted of the incisors 1–15 and group B of the incisors 16–30. Four dentin samples (a–d) were derived from each incisor creating 60 dentin samples in each group. The procedure of the sample preparation is already demonstrated in detail in an earlier study [10]. The 60 dentin samples in each group were divided into four subgroups (A1–A4 and B1–B4). Each subgroup consisted of 15 dentin samples which were derived from 15 different incisors as shown in Table 1. The samples were then ground with water-cooled silicon carbide paper (Tegramin-30, Struers, Copenhagen, Denmark) in an automatic grinding machine as follows: Subgroups A1 and B1 were ground with 1200-grit paper (5 s), subgroups A2 and B2 were ground with 1200- and 2000-grit paper (5, 10 s respectively), subgroups A3 and B3 were ground with 1200-, 2000- and 4000-grit paper (5, 10, 30 s, respectively), subgroups A4 and B4 were ground with 1200-, 2000-, 4000-grit paper (5, 10, 30 s, respectively) and then subjected to 1000 brushing strokes (120 strokes/min, 2.5 N) using a slurry of Elmex Kariesschutz (Colagte-Palmilive, Swidnica, Poland) toothpaste. The grinding was conducted at 5 N pressure load and under constant water cooling. The slurry was prepared by mixing one part of Elmex toothpaste with two parts of artificial saliva [14] for five minutes. After this pretreatment, reference areas within the dentin surface were covered using an adhesive tape and the baseline profiles of all samples were recorded with a stylus profilometer (MFW-250, Perthometer S2; Mahr, Göttingen, Germany). Five profiles with a distance of 250 µm were recorded per sample. The detailed procedure of the profilometric analysis is already described in a previous study [15]. The profilometric profiles were recorded under wet conditions to prevent the dentin samples from desiccation. The samples underwent a brushing sequence in a custom-made 6-place-cross-brushing-machine using medium-hard standard toothbrushes (Paro M43, Esro; Thalwil, Switzerland). The load applied by the toothbrush on the samples was set at 2.5 N. The brushing sequence lasted for 25 min at a brushing frequency of 120 strokes/min. Samples in group A were brushed using a slurry of an abrasive with an RDA value of 85 (Sident 2480-1, Evonik industries, Hanau-Wolfgang, Germany). Samples in Group B were brushed using a slurry of an abrasive with an RDA value of 174 (Zeodent 103, Evonik industries, Hanau-Wolfgang, Germany). The slurries were prepared by mixing 90 g of the respective abrasive with 450 g of glycerine and 0.45 g of a silicon anti-foam agent for five minutes and added afterwards to the brushing chamber. Fresh slurry was added with 5-min intervals. After the brushing sequence, final profiles were recorded. A custom-made jig was used to ensure the exact repositioning of the samples into the profilometer. Table 1 summarises the multifactorial study design (two levels of slurry abrasivity and four levels of pretreatment procedure).

**Table 1:** Study design

30 bovine permanent incisors							
Group A (incisors 1 to 15)				Group B (incisors 16 to 30)			
4 dentin samples (a to d) from each incisor n = 60				4 dentin samples (a to d) from each incisor n = 60			
A1 n = 15 (samples -a- of the incisors 1 to 15)	A2 n = 15 (Samples -b- of the incisors 1 to 15)	A3 n = 15 (Samples -c- of the incisors 1 to 15)	A4 n = 15 (Samples -d- of the incisors 1 to 15)	B1 n = 15 (Samples -a- of the incisors 16 to 30)	B2 n = 15 (Samples -b- of the incisors 16 to 30)	B3 n = 15 (Samples -c- of the incisors 16 to 30)	B4 n = 15 (Samples -d- of the incisors 16 to 30)
Pretreatment of the samples as follows:							
1200 grit	1200 grit + 2000 grit	1200 grit + 2000 grit + 4000 grit	1200 grit + 2000 grit + 4000 grit + 1000 brushing strokes	1200 grit	1200 grit + 2000 grit	1200 grit + 2000 grit + 4000 grit	1200 grit + 2000 grit + 4000 grit + 1000 brushing strokes
Recording of baseline profiles							
Brushing sequence (25 min, 120 strokes/min)							
RDA-85 Slurry (Sident 2480-1)				RDA-174 Slurry (Zeodent 103)			
Recording of final profiles							

### Statistical analysis

For each group, mean values and standard deviations of the abrasive dentin wear (µm) in regard to the different pretreatment procedures (1200-grit paper, 1200- + 2000-grit paper, 1200- + 2000- + 4000-grit paper, 1200- + 2000- + 4000-grit paper + 1000 brushing strokes) were calculated and analysed using repeated measures one-way ANOVA. Subgroups in each group were compared pairwise and the resulting *p* value was corrected after Tukey. Data was analysed using the statistical program R (The R Foundation for Statistical Computing; Vienna, Austria; www.R-project.org).

## Results

### Group A (RDA 85)

**Fig. 1:**
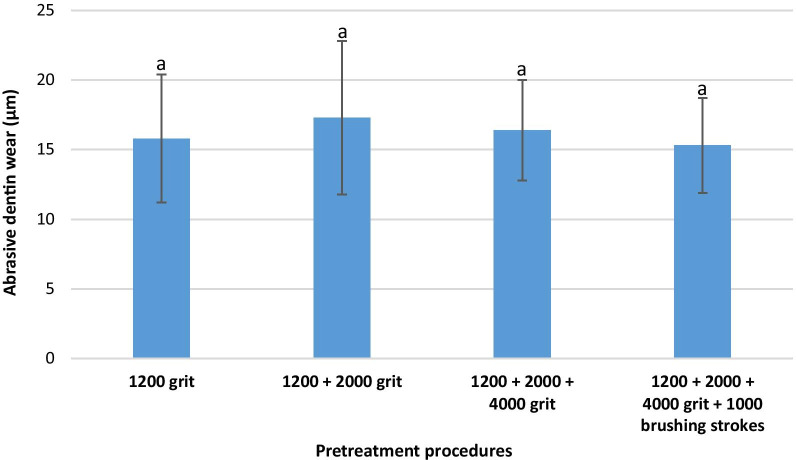
Abrasive dentin wear and standard deviation in group A (RDA 85). Same small letters indicate no significant statistical difference

The mean abrasive dentin wear (± standard deviation) was calculated in each subgroup (each different pretreatment procedure) as follows: 1200 grit: 15.8 ± 4.6 µm, 1200 + 2000 grit: 17.3 ± 5.5 µm, 1200 + 2000 + 4000 grit: 16.4 ± 3.6 µm, 1200 + 2000 + 4000 grit + 1000 brushing strokes: 15.3 ± 3.4 µm. The differences between all subgroups were not statistically significant (*p *˃ 0.05). Figure 1 demonstrates the calculated abrasive dentin wear in group A.

### Group B (RDA 174)

**Fig. 2:**
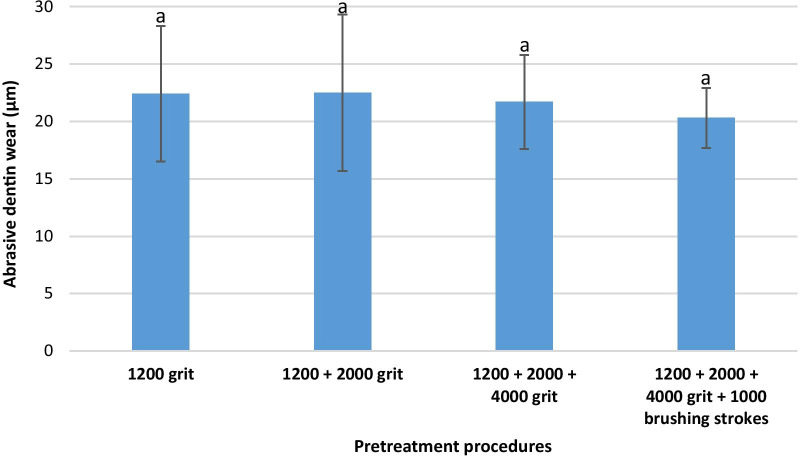
Abrasive dentin wear and standard deviation in group B (RDA 174). Same small letters indicate no significant statistical difference

The mean abrasive dentin wear (± standard deviation) was calculated in each subgroup (each different pretreatment procedure) as follows: 1200 grit: 22.4 ± 5.9 µm, 1200 + 2000 grit: 22.5 ± 6.8 µm, 1200 + 2000 + 4000 grit: 21.7 ± 4.1 µm, 1200 + 2000 + 4000 grit and 1000 brushing strokes: 20.3 ± 2.6 µm. The differences between all subgroups were not statistically significant (*p* ˃ 0.05). Figure 2 demonstrates the calculated abrasive dentin wear in group B.

## Discussion

Dentin samples used in abrasion studies are usually pretreated before subjected to the abrasion sequence. This pretreatment helps reaching a standard baseline situation for all the samples. Different pretreatment procedures of dentin samples have been reported in the literature. The effect of different pretreatment procedures on the resulting abrasive dentin wear has not yet been investigated. This study was therefore conducted to investigate whether different pretreatment procedures of dentin samples would influence the resulting abrasive dentin wear. The findings of this study might contribute to explaining the different abrasivity values measured in different laboratories and/or simplify the complex pretreatment procedures carried out in some laboratories if found not necessary.

Bovine dentin was used in this study. Four samples were extracted from each tooth and divided among the four subgroups to provide a certain harmony in the baseline properties in each subgroup. Bovine teeth have larger surfaces than human ones and allow the extraction of more samples from a single tooth. The suitability of bovine dentin in abrasion studies as an alternative to human dentin was reported in an earlier study [16]. The dentin samples were brushed for 25 min in both groups, which is a standard brushing time in RDA method [17].

Two different slurries made of two different abrasives were used in this study. The slurry in group B had twice the RDA value of the slurry in group A (174 vs. 85). Conducting this study using only the RDA-85 slurry (only group A) would also have helped answering the study question. However, as the ISO-regulations for toothpastes set the maximum allowed RDA value at 250, the authors considered also using a slurry with high RDA value to observe the findings under such conditions. To prepare the RDA-174 slurry, a different abrasive was used rather than using twice the amount of RDA-85 abrasive, which might have led to an exaggerated viscosity of the slurry, and hence alter its abrasivity.

In this study, dentin samples with different pretreatment procedures showed the same amount of abrasive dentin wear. This applies regardless of the RDA value of the slurry with which the samples were brushed. This finding could be considered as unexpected. As the resulting abrasive dentin wear depends on many factors, one would expect that the property of the very surface, which is being directly rubbed with the abrasives, might as well play a role, and somehow alter the way the abrasives interact with the surface. However, it could be speculated that the differences between the subgroups after various pretreatment procedures were so little, that the relative high abrasive wear might have masked them. The fact that the abrasive wear was recorded using a stylus (contact) profilometer in this study should be kept in mind. An optical profilometer might be affected by different surface properties (e.g., gloss) of the sample which might result in different readings. It might be advisable to investigate this possible effect in further studies.

Another interesting finding in this study is the resulting abrasive dentin wear value between groups A and B. As mentioned above, samples in group B were brushed with an abrasive slurry with twice higher RDA value than samples in group A (174 vs. 85). However, the mean calculated profilometric abrasivity were approximately 16 µm in group A and 22 µm for group B. This discrepancy in measured abrasive dentin wear between radiotracer method (RDA) and profilometric method has also been observed in other studies [18–20]. This finding should however not be over-interpreted, as samples in both groups were not derived from the same teeth and were not compared to each other statistically. Further studies that aim to explore the discrepancies between both methods might be advisable.

## Conclusions

Within the limits of this study, different pretreatment procedures of dentin samples do not result in different abrasive wear after 25 min brushing time independent of the RDA value of the employed slurry. The null hypothesis cannot be rejected. This finding might be useful for the different laboratories which carry out abrasion studies and use the same pretreatment procedures described here. Simplifying the complex pretreatment steps could have time-saving and economic benefits.

## Data Availability

The datasets used and/or analysed during the current study are available from the corresponding author on reasonable request.

## Abbreviation

RDA: Relative dentine abrasivity
