# Correction to: Effect of dentin pretreatment on the resulting abrasive dentin wear

**DOI:** 10.1186/s12903-021-01672-3

**Published:** 2021-06-24

**Authors:** Blend Hamza, Marina Kazimi, Philipp Körner, Thomas Attin, Florian Just Wegehaupt

**Affiliations:** 1 Clinic of Orthodontics and Pediatric Dentistry, Center of Dental Medicine, University of Zurich, Plattenstrasse 11, 8032 Zurich, Switzerland; 2 Clinic of Conservative and Preventive Dentistry, Center of Dental Medicine, University of Zurich, Zurich, Switzerland

## Correction to:BMC Oral Health (2021) 21:295 https://doi.org/10.1186/s12903-021-01648-3

After publication of the original article [1], an error was identified in the authors’ affiliations list.

The correct affiliations list is given below:

Blend Hamza^1,*^, Marina Kazimi^2^, Philipp Körner^2^, Thomas Attin^2^ and Florian Just Wegehaupt^2^.

^1^Clinic of Orthodontics and Pediatric Dentistry, Center of Dental Medicine, University of Zurich, Plattenstrasse 11, 8032 Zurich, Switzerland;

^2^Clinic of Conservative and Preventive Dentistry, Center of Dental Medicine, University of Zurich, Zurich, Switzerland.

The original article has been corrected.
